# Genome Sequences of Multidrug-Resistant *Salmonella enterica* Serovar Paratyphi B (dT+) and Heidelberg Strains from the Colombian Poultry Chain

**DOI:** 10.1128/genomeA.01265-15

**Published:** 2015-10-22

**Authors:** Pilar Donado-Godoy, Johan F. Bernal, Fernando Rodríguez, Yolanda Gomez, Richa Agarwala, David Landsman, Leonardo Mariño-Ramírez

**Affiliations:** a Corporación Colombiana de Investigación Agropecuaria (CORPOICA), Centro de Investigación Tibaitatá, Cundinamarca, Colombia; b National Center for Biotechnology Information, National Library of Medicine, National Institutes of Health, Bethesda, Maryland, USA

## Abstract

*Salmonella enterica* is a pathogen of significant public health importance that is frequently associated with foodborne illness. We report the whole-genome sequences of four multidrug-resistant *Salmonella enterica* serovar Paratyphi B and Heidelberg strains, isolated from the Colombian poultry chain. The isolates contain a variety of antimicrobial resistance genes for aminoglycosides, β-lactams, fluoroquinolones, sulfonamides, tetracycline, and trimethoprim.

## GENOME ANNOUNCEMENT

*Salmonella* spp. continue to be one of the most important causes of foodborne gastroenteritis globally, affecting mainly infants under 1 year and children between 1 and 4 years of age (1). *Salmonella* serovars Paratyphi B and Heidelberg have been determined to be causative of morbidity and mortality in humans (2, 3). Recently, *Salmonella enterica* serovar Paratyphi B and Heidelberg multidrug-resistant (MDR) strains have also been reported as the two most prevalent serovars in Colombian poultry (4–7). The four *Salmonella* isolates described here are part of a comprehensive prevalence survey from the Colombian Integrated Program for Antimicrobial Resistance Surveillance (COIPARS) (8) and were recovered from retail stores, slaughterhouses, and cecal contents during 2012 to 2013 in important poultry production regions (Cundinamarca and Santander) as well as Bogotá, Colombia. These *Salmonella* isolates are resistant to several families of antibiotics including β-lactams, quinolones, fluoroquinolones, aminoglucosides, tetracyclines, nitrofurantoins, in addition to folate pathways and β-lactamase inhibitors.

Here, we report the whole-genome sequences of four *Salmonella enterica* serovar Heidelberg (FSAN332CC, UG1286CA) and Paratyphi B (FCUN156CA, FSAN236CA) strains. Genomic DNA from each strain was isolated from overnight cultures using the PureLink Genomic DNA minikit (Invitrogen) and DNA libraries were prepared using the Nextera XT DNA sample preparation kit (Illumina). The libraries were prepared according to the manufacturer’s instructions and sequenced on an Illumina MiSeq instrument with 2 × 250-bp paired-end reads, according to standard Illumina protocols. The four genomes were assembled using a reference-guided assembler ARGO, developed at NCBI, and a *de novo* assembler SPAdes (9) to create initial assemblies of the reads. These were combined to produce the final assembly using a conservative process that incorporates pieces of *de novo* assembly in the reference-guided assembly. The reference genome GenBank accessions for the assembly were CP000886 (Paratyphi B) and CP001120 (Heidelberg). The genome sequences of strains FSAN332CC, UG1286CA, FCUN156CA, and FSAN236CA consisted of 192, 58, 56, and 58 contigs, respectively, yielding total sequences for each isolate of 5,160,508, 5,135,585, 4,900,493, and 4,996,395 bp, respectively. The overall G+C content for the isolates was determined to be 52.5%. Sequences were annotated using the NCBI Prokaryotic Genomes Automatic Annotation Pipeline (PGAAP) and have been deposited at GenBank. The results of the genome annotation presenting the number of genes, coding sequences (CDSs), rRNA, tRNA, CRISPR arrays, pseudogenes, and noncoding RNA (ncRNA) are summarized in Table 1.

**TABLE 1:** Genome annotation statistics

NCBI BioSample	No. of genes	No. of CDSs	No. of pseudogenes	No. of CRISPR arrays	No. of rRNAs	No. of tRNAs	No. of ncRNAs	GenBank accession no.
SAMN03842020	5,104	4,901	74	2	24	84	21	LIKS00000000
SAMN03842018	5,026	4,832	64	2	24	84	22	LIKR00000000
SAMN03842017	4,775	4,602	79	2	8	73	13	LIKQ00000000
SAMN03842015	4,883	4,713	76	3	8	73	13	LIKP00000000

A search for resistance-associated genes present in all isolates was performed using ResFinder (v2.1) (10) with default parameters. We found antimicrobial resistance genes for aminoglycosides [*strA, aph*(*3′*)-*Ia, aadA1*, and *strB*], β-lactams (*blaCMY-2*, *blaCTX-M-2*, and *blaTEM-1B*), fluoroquinolones (*QnrB19*), sulfonamides (*sul1* and *sul2*), tetracycline [*tet*(*A*)], and trimethoprim (dfrA1,dfrA7). Future comparative analyses will advance national surveillance programs and our understanding of genome evolution and multidrug resistance in *Salmonella*.

### Nucleotide sequence accession numbers.

This whole-genome shotgun project has been deposited in DDBJ/EMBL/GenBank under the accession numbers listed in Table 1. The version described in this paper is the first version. The BioProject accession is PRJNA289090.
